# Victims of Known and Unknown Cyberstalkers: A Questionnaire Survey in an Italian Sample

**DOI:** 10.3390/ijerph19084883

**Published:** 2022-04-17

**Authors:** Tatiana Begotti, Mariano Alex Ghigo, Daniela Acquadro Maran

**Affiliations:** 1 Department of Psychology, Università di Torino, 10124 Torino, Italy; tatiana.begotti@unito.it (T.B.); mariano.ghigo@edu.unito.it (M.A.G.); 2 WOW-Work and Organizational Wellbeing Research Group, 10124 Torino, Italy

**Keywords:** online harassment, anxiety symptoms, depressive symptoms, coping strategy

## Abstract

Cyberstalking is a behavior in which an individual, group, or organization uses information technology to harass one or more people, with possible consequences for the victims. The purpose of this study was to analyze the effects of cyberstalking in terms of physical and emotional consequences, depression, anxiety symptoms, attitudes toward telling of cyberstalking experiences, and coping strategies, comparing young adult victims of known cyberstalkers with those harassed by strangers. A self-administered questionnaire was completed by 689 individuals. Of these, 242 victims were included in the analysis: 115 victims of unknown (UC) and 127 of known cyberstalkers (KC). The results emphasize that victims of KC more often reported fatigue as a physical symptom and sadness and lack of trust in others as emotional symptoms. In addition, scores for depressive symptoms and anxiety did not differ significantly between the two groups, whereas significantly higher scores for trait anxiety were found among victims of KC. Finally, victims of KC were significantly more inclined to use alcohol and drugs, reduce social contact with friends, buy a weapon, and try to reason with the cyberstalker, while victims of UC were more inclined to block online contact and ask a social network administrator to intervene. Implications of the findings were discussed, such as the need to intervene immediately and to promote victims’ ability to seek help.

## 1. Introduction

Cyberstalking was defined by Bocij et al. [1] as behavior in which an individual, group, or organization uses information technology to harass one or more individuals. This can include online contact, online harassment, unwanted sexual advances, threats of violence, and identity fraud. This phenomenon is a form of cyberbullying, both of which are characterized by repeated aggressive online behavior towards the victim, who is accessible anytime and anywhere [2,3,4,5]. The difference lies in the motives of the harassment. The cyberbullies are not so much interested in the person they are bullying but rather in the act of bullying itself, the opportunity to assert their power over the victim in general. The cyberbullies also need an audience, onlookers who applaud their behavior. The cyberstalker, on the other hand, harasses the victim with the intention of restoring or establishing a love relationship. His/her attention is focused only on him/her, and he/she would be visible without showing his/her true identity. As Wilson et al. [6] point out, cyberstalking is characterized by the presence of one or more unwanted behaviors that can cause psychological and emotional consequences for victims.

It has been estimated that between 20 and 40 percent of online users worldwide have experienced at least one cyberstalking behavior [7]. Reyns et al.’s [8] findings on a sample of 974 U.S. students indicate that 40% have experienced cyberstalking. Maple et al. [9] found in a sample of 353 participants that 324 of them were victims. Their study found that in 22% of the cases, the perpetrators were unknown, in 21% of the cases, they were acquaintances, and in 18%, they were people who happened to be known. The remaining portion of the sample had an intimate relationship with the cyberstalkers. In a qualitative study by Mcfarlane and Bocji [10] involving 24 victims, it was found that in 22% of the cases, the perpetrator was a stranger, 33% was a person they had recently met online, 16.7% was an acquaintance, 12% was a colleague, and 12% was a former confidant. 

These data contrast with some previous research. For example, a study by Working to Halt Online Abuse (WHOA [11]) in the U.S. found that among 220 self-identified victims, in 61% of cases, the cyberstalker had a previous relationship with the victim and in 26.5% of cases was a former significant other. In the research conducted by Reno [12], the existence of a previous relationship appears to be the primary cause of cyberstalking. In the research conducted by Bainbridge [13], in which 100 subjects participated, 50% of the victims were harassed by a friend or acquaintance, 30% by a stranger; 65% of them were threatened or harassed online (see also [14]). In addition, the study by Dressing et al. [15], which involved 6379 people who completed an online questionnaire about cyberstalking, found that 29.3% of victims were harassed by an ex-partner. Interestingly, the results of Ahlgrim and Terrance’s [16] study showed that cyberstalking by a stranger was perceived as more severe than when the cyberstalker was a former intimate partner. 

### 1.1. Cyberstalking Behaviors

The excessive use of technology-enabled communication platforms (such as social media) by individuals has led scholars to recognize the increasing instances of cyberstalking [17]. Online forms of interpersonal violence are becoming a fact of life for adults of all ages [18]. Cyberstalking can affect all age groups, with a particular focus on young people, who can be harassed and monitored online in a wide variety of ways [19]. About behavior, Fischer et al. [20] reported that 24.7% of 4446 respondents had been harassed or threatened by email. WHOA [11] has shown that in the 234 cases of cyberstalking reported in the last nine years, most harassment begins through initial contact via email. Email allows perpetrators to send repeated harassment, threats, messages with obscene images, videos, and audio files. Cyberstalkers might use special programs that enable the repeated and continuous sending of harassing emails. In addition, these services allow perpetrators to remain completely anonymous and untraceable to avoid court problems [12]. Emails are also used (although to a lesser extent) to send viruses that can damage the victim’s computer [21]. Another medium used by cyberstalkers is the chat room. In this way, they can get in touch with an often unknown victim. Initially, they turn out to be nice and friendly. Once they realize that the relationship is at risk (because the interlocutor tries to reduce contact or shows disinterest), they use behaviors to discredit the victim, such as finding out his or her personal information through identity theft [22]. Cyberstalking can also take place in forums, allowing a large online audience to participate. Posting embarrassing and humiliating messages about the victim on the Internet is referred to as “cybersmearing” (translated as cyberdefamation). The most common form involves posting sexual images or videos in which the victim is insulted [23]. A study conducted by Finn [24] on a sample of 339 college students at the College of New Hampshire confirmed that harassment occurred through emails and messages. Specifically, 16.2% of the participants were harassed through emails and 19.3% through instant messages from strangers. A total of 58.7% of the sample described that the most common content in the emails they received was pornographic. The National Center for Cyberstalking Research [9] survey, which included 353 participants, found that 74.4% of them reported multiple avenues of harassment. Social media was the most common, followed by email and text messaging (SMS).

### 1.2. Physical and Emotive Consequences

As Wilson et al. [6] noted, the phenomenon can have emotional and physical consequences for victims. Many victims suffer from insomnia, eating disorders, nightmares, hypervigilance, anxiety and depressive symptoms, and fear for their own safety [25]. Hypervigilance is often associated with PTSD (post-traumatic stress disorder) [26]. Maple et al. [9] showed that 80.4% of the 324 cyberstalking victims surveyed suffered both physical and social consequences, such as damage to their reputation. Their results show that 94.1% of them suffered from stress. The cyberstalking campaign had consequences on their working life: In 73.3% of the cases, there were negative effects on their work, e.g., they had to change jobs or were fired. The victims also changed their social relationships as a result of the harassment. The most important consequences were the loss of friends (39.6% for women, 32.6% for men) and the abandonment of social activities (54.7% for men, 43.1% for women). Cyberstalking also results in loss of money due to expenses for lawyers, securing housing, moving, or counseling/therapy. The study by Dressing et al. [15] shows that 68.2% of the victims lost trust in others, 64.2% reported sleep disturbances, 54.9% felt anger, 49.6% had difficulty meeting new people, and 48.1% had concentration problems. Only 2.5% of the sample reported no psychosomatic consequences. In addition, research on past relationships and consequences in victims had shown that victims experienced more negative feelings when the perpetrator was a confidant or acquaintance than a stranger [27,28,29]. In Acquadro Maran and Begotti’s [30] study of 107 Italian victims of cyberstalking, the most commonly reported physical symptoms included sleep disturbances, fatigue, panic attacks, headaches, feeling weak, and nausea. Emotional symptoms included anger, irritation, sadness, confusion, and anxiety. In addition, another study [31] showed that the mean score of depressive symptoms was significantly higher in victims of cyberstalking than in non-victims, while the mean scores of state and trait anxiety symptoms were not significantly different in victims and non-victims of cyberstalking.

### 1.3. Telling Someone and Coping Strategies

Victims generally tend not to report their experiences [14] and are more likely to seek help informally than formally. The results of the research conducted by Finn [24] showed that 21% of victims did not report the case of cyberstalking; 37.5% did not think the problem was very important, 19.5% ignored the facts, 19.5% coped with the behavior without help, and 12.5% did not know whom to ask for help. Maple [9] showed that 53.4% of the 324 victims of cyberstalking reported the harassment to the police, 59% did not respond to the cyberstalker, 46.3% turned off the PC or smartphone, 46% tried to confront the cyberstalker, 35% left the social network, 33.6% changed their email address, and 32.4% reported to the website administrator. In the study conducted by Bainbridge [13], the results showed that 80% of victims asked friends and family for help, while 10% asked school administrators. No one asked the police for help. The latter data is consistent with what Reno [12] found regarding cyberstalking complaints to the police. In Fissel’s [32,33] study involving 477 cyberstalking victims, 43.8% of them sought informal help (e.g., friends), and 14.5% sought formal help (e.g., the police). In a study by Alexy et al. [34], it was found that the largest proportion of victims (75%) told friends about their experience, 54% told parents, while 11% told no one. Telling someone about the experience of victimization is one of the coping strategies. The choice of an appropriate coping strategy could determine the termination of the stalking campaign [35]. The results of Amar and Alexy’s study [36] show that victims use several strategies to stop the cyberstalker. Among these strategies, Podanà and Imriskova [37] categorized three types of general strategies: proactive behavior, avoidance tactics, and passivity (see also [38,39]). Proactive behavior included a coping strategy aimed at resolving the situation, such as asking a social network administrator to intervene or reporting the victimization to the police. Avoidance tactics are strategies used by victims to cope with the situation by changing their usual activities and routines, such as limiting their use of the Internet. Finally, through passivity strategies, victims try to ignore the cyberstalker, for example, by distracting themselves with other activities. In the study by Podanà and Imríšková [37], it was found that 47% of 147 victims reported proactive behavior, 30% reported avoidance tactics, and the rest of the sample reported the passivity strategy. In the meta-analysis by Littleton et al. [40], an association between passivity strategies and negative outcomes, such as stress and poorer health, was found. In addition, Kraaij et al. [41] found that both passive strategies and proactive behaviors were associated with higher depression and anxiety symptoms. As Begotti and Acquadro Maran [25] pointed out, a possible trigger was the emotional fatigue of using strategies that require ignoring the misbehavior or, on the contrary, hitting the cyberstalker. In their study, the results showed that victims tended to use avoidance tactics rather than passivity strategies and proactive behaviors. As Reyns et al. [8] noted, victims of known cyberstalkers (e.g., friends, acquaintances, ex-partners) were more inclined to use a variety of coping strategies (consisting of telling someone). This is because the information the perpetrator has is more comprehensive and includes various aspects of the person’s life, not just those available online. The stranger/unknown, on the other hand, only has what the victim posts on social media. 

In Italy, stalking has been a criminal offense since 2009 (**Criminal** Code, Article 612 bis, 2009, cit. in [30]). The law states the following: “Unless the act is recognized as a more serious offense, it is a crime punishable by imprisonment of six months to four years to continuously threaten or harass another person to such an extent that a serious, persistent state of anxiety or fear is created. or instilling in the victim(s) a well-founded fear for his or her own safety or for the safety of relatives or other persons connected to the victim(s) by kinship or emotional ties, or forcing the victim(s) to change his or her living habits.” In the Italian legal system, online behaviors such as defamation, identity fraud, and hacking are considered an extension of stalking [42]. In Italy, on average, 18.7% of men and 21.5% of women [43,44] are victims of stalking. However, to the best of our knowledge, there is no research on the characteristics of the behavior committed by known and unknown cyberstalkers, as well as on the consequences and coping strategies. The purpose of this study was to analyze the impact of cyberstalking in terms of physical and emotional consequences, depression, anxiety symptoms, attitudes toward telling someone about the cyberstalking experience, and coping strategies, comparing victims of known cyberstalkers (e.g., friends, acquaintances, ex-partners) with those victimized by strangers. The study examined a sample of young adults because, as suggested by Dressing et al. [15], they are more at risk of becoming victims of cyberstalking than other individuals. Based on the literature reviewed, the hypotheses were as follows: 

(1) Victims of known cyberstalker report more physical and emotional symptoms, anxiety, and depression than victims of unknown perpetrators.

(2) Victims of known cyberstalker are more inclined to tell someone about their experience and to use a greater variety of coping strategies.

## 2. Materials and Methods

### 2.1. Participants

A self-administered questionnaire was distributed to over 700 young adults in Italy. The snowball sampling approach was used. We contacted university students attending different courses. Then, we asked them to recruit subjects from among their acquaintances. The instrument was completed by 689 individuals. A total of 320 participants (46.4%) reported having been victims of at least one form of cyberstalking. A total of 78 individuals were excluded from the analysis because they had been harassed by both known and unknown perpetrators. Of the remaining 242 participants, 74% were female and 26% were male. They were between 18 and 30 years old (*M* = 23, *SD* = 2.7). The majority were single (56%), 36.6% were engaged, and 5.2% were cohabiting; the remaining participants did not answer the question or used the “other” category.

### 2.2. Measures

#### 2.2.1. Prevalence of Cyberstalking

The prevalence of cyberstalking was measured using the questionnaire developed by Reyns et al. [8]. Items were adapted for use with an Italian sample by translating them from British English and then back-translating them. The questionnaire has been used in previous research on cyberstalking in Italy [25,30,31]. The respondents were asked whether they had experienced behaviors that could be defined as cyberstalking, specifically: Contacting (“Has anyone ever contacted or attempted to contact you online on more than one occasion after you asked/said to stop?”), Harassment (“Has anyone ever harassed or attempted to harass you online on more than one occasion?”), unwanted sexual advances (“Has anyone made unwanted sexual advances to you online on more than one occasion?”), threats of violence (“Has anyone spoken to you violently or threatened to physically hurt you online on more than one occasion?”), or identity fraud (“Has anyone impersonated you online without your permission?”) (possible response options: yes/no). For each type of behavior, the participants indicated the relationship between victim and perpetrator (possible responses: partner/ex-partner, friend/acquaintance, stranger). For this study, the partner/ex-partner and friend/acquaintance categories were combined into a single category called known cyberstalker (KC), which was compared to unknown cyberstalker (UC). 

#### 2.2.2. Physical and Emotional Symptoms

The consequences of the cyberstalking experience were measured using 23 items from the Italian version of the Stalking Questionnaire [45]. Twelve items examined physical symptoms (e.g., weight change, loss of appetite, sleep disturbances; yes/no responses), and 11 items examined emotional consequences (e.g., sadness, anger, confusion; yes/no responses). 

#### 2.2.3. Depressive Symptoms

Depressive symptoms were measured with the abbreviated Beck Depression Inventory (BDI) [46,47]. It consists of 13 items that can be used to classify symptoms and determine different levels of severity: no or minimal depression (scores 0–4), mild depression (5–7), moderate depression (8–15), or severe depression (>15) (in this study, Cronbach’s α was 0.87, which indicated excellent internal consistency).

#### 2.2.4. Anxiety Symptoms

The State-Trait Anxiety Inventory was used to measure anxiety symptoms [48,49]. It consists of two forms (STAI-Y1 and STAI-Y2; 20 items each) that assess how participants feel in the present moment and how they feel most of the time (for state anxiety and trait anxiety, respectively). Total scores can range from 20 to 80, with 40 being the threshold score considered indicative of anxiety symptoms. The rating scale has different severity levels: from 40 to 50 mild, from 51 to 60 moderate, and >60 severe. The Cronbach’s α values in this study were 0.93 and 0.92, respectively, which indicated excellent internal consistency. 

#### 2.2.5. Telling Someone about the Cyberstalking Experience and Coping Strategies

The measures used to examine participants’ attitudes toward telling someone about their cyberstalking experience and different coping strategies were taken from the Italian version of the Stalking Questionnaire [45]. Potential confidants included police, parents/family, friends, partners, physicians, and psychotherapists (6 items; yes/no responses). Coping strategies were measured using 15 items. The different coping strategies included items on proactive behavior (e.g., gather evidence; try to contact and reason with the cyberstalker), avoidance tactics (e.g., limit Internet use; stop online contact), and passivity (e.g., increase abuse of alcohol; increase use of psychotropic substances) (yes/no responses).

### 2.3. Ethical Statement and Procedure

All ethical guidelines required for the conduct of research involving human subjects were followed, including compliance with legal requirements in Italy. This research project was approved by the local ethics committee (N.277326/2017). The cover sheet clearly explained the research objective, the voluntary nature of participation, the anonymity of the data, and the elaboration of the results. Thus, returning the questionnaires signified consent. The questionnaire was administered individually in paper form, and it was returned immediately after compilation. The data were collected by four research assistants who had been previously trained by the researchers. Together with the questionnaire, each participant received an information letter and a consent form. It took approximately 20 min to complete the questionnaire. Respondents participated in the study voluntarily and received no compensation (or extra credit) for their participation.

### 2.4. Data Analysis 

The data were processed with SPSS version 28 (IBM Corp., Armonk, NY, USA). To assess the significance of differences between victims of unknown and known cyberstalkers, *χ*^2^ tests were used. The phi value was calculated to estimate the effect size. The data were also analyzed using ANOVA to measure differences between UC and KC. Eta squared was calculated to estimate the effect size. 

## 3. Results

### 3.1. Information about the Victims of Cyberstalking

Regarding the specific behaviors related to cyberstalking, of the 242 victims included in the study, 117 individuals (48.3%) reported being victims of cyberstalking through online contact, 73 (30.2%) through online harassment, 102 (42.1%) through unwanted sexual advances online, 43 (17.8%) through online threats of violence, and 41 (16.9%) through online identity fraud. More detailed information about the distribution of the different behaviors in terms of the gender of the victim or the perpetrator and the relationship between the two can be found in Table 1. It should be noted that victims indicated the gender of the perpetrator on the basis of the self-identification: some of the perpetrators could sign with a male name and be a woman.

### 3.2. Physical and Emotional Symptoms

In many cases, victims reported negative consequences related to their cyberstalking experience. The comparison between victims of strangers (unknown cyberstalker, hereafter UC; *n* = 115) and victims of friends/acquaintances, partners/ex-partners (known cyberstalker, hereafter KC; *n* = 127) in terms of physical and emotional symptoms was made considering all forms of cyberstalking as a whole, without distinguishing between the different behaviors. To compare the victims of unknown and known perpetrators, the chi-square test was used (Table 2). It should be noted that all symptoms (such as weight change) were assessed by self-perception.

In general, victims of KC more often reported tiredness as a physical symptom and sadness and lack of confidence in others as emotional symptoms than victims of UC.

### 3.3. Depressive and Anxiety Symptoms

Overall, 135 subjects in the sample (61%) had a minimal score on the Beck Depression Inventory (BDI). The remaining participants had a score on the scale corresponding to mild (*n* = 35; 16%), moderate (*n* = 40; 18%), or severe (*n* = 11; 5%) depression (221 subjects met all items of the BDI). Regarding the presence of anxiety symptoms, 90 subjects (41.9%) scored below 40 on the first part of the STAI-Y1, which is considered the threshold for clinically significant anxiety symptoms. The remainder of the sample exhibited mild (*n* = 75, 34.9%), moderate (*n* = 31, 14.4%), and severe (*n* = 19, 8.8%) anxiety (215 subjects met all items of the STAI-Y1). In addition, 86 subjects (38%) in this study reported a score below 40 on the second part of the STAI-Y2. The rest of the sample had mild (*n* = 83, 37%), moderate (*n* = 41, 18%), and high (*n* = 16, 7%) trait anxiety scores (226 subjects fulfilled all items of the STAI-Y2). A one-way ANOVA was conducted to compare the victims of UC and KC, introducing the BDI scale and the two scores on the STAI-Y1 and Y2 scales as dependent variables (Table 3). 

As can be seen in Table 3, the scores for depressive and state anxiety symptoms did not differ significantly between the groups affected by UC and KC. However, it was found that the scores for trait anxiety of the victims of KC were, on average, significantly higher than those of the victims of UC.

### 3.4. Telling Someone about the Cyberstalking Experience and Coping Strategies 

Most victims of cyberstalking reported that they told someone about their experience (*n* = 171, 70.7%), with a higher tendency to talk about it with their friends (*n* = 153, 63.2%). It was also found that, specifically for victims who talked to their partner, there was a statistically significant difference between victims of UC (*n* = 36, 31.3%) and KC (*n* = 25, 19.7%) (*Χ*^2^ = 7.984; *p* = 0.005; *ϕ* = −0.221). No other statistically significant differences were found for other potential confidants (police, parents/family, friends, physicians, and psychotherapists) (Table 4). 

The list of coping strategies considered and the prevalence of their use in both groups are shown in Table 5.

The table shows that victims of KC have a significantly higher tendency to use more than UC victims-specific coping strategies, such as increasing the use of alcohol and drugs, reducing social contact with friends, buying a weapon, and trying to reason with the cyberstalker. At the same time, there are some strategies that are more likely to be used by UC victims than KC victims, such as blocking online contact and asking the administrator of a social network to intervene.

## 4. Discussion

The aim of this work was to analyze the consequences and coping strategies used by young adults, self-identified victims of cyberstalking. A comparison was made between victims of partners, ex-partners, acquaintances, friends, etc., and victims of strangers. For the purposes of this study, two types of cyberstalkers were distinguished: Known (i.e., there was a prior relationship between victim and perpetrator) and unknown cyberstalkers (i.e., there was no prior relationship between victim and perpetrator). Overall, the prevalence of victimization was 46.4%, similar to that described by Reyns et al. [8]. Online contact was the most common behavior reported by victims. Victims were female in 72% of cases, while the perpetrator was male in 78% of cases. Stranger perpetrators were reported by 35% of victims in this type of misconduct. The largest proportion of victims described the cyberstalker as KC (65%), confirming the findings of WHOA [11] and Berry and Bainbridge [13,14]. Regarding consequences, the BDI indicated a minimal score for depressive symptoms in most participants. However, 39% of participants reported that symptoms ranged from mild to severe depression. Therefore, it is important to intervene immediately and provide psychological support to victims. As Kernig et al. [50] noted, victims for whom the abuse had ended were less likely to report depression. In addition, Wright [51] highlighted that for victims of cyberstalking, depressive symptoms were related to lower levels of perceived social support.

This could mean that coping strategies involving sharing the experience with others could be useful in managing the negative feelings caused by the misconduct. The coping strategies most frequently mentioned by victims were gathering evidence, increasing social contact, and reducing Internet use. The results show that most victims reported that they told someone about their experience, especially friends (153, 63.2%). UC victims more often indicated the strategy of telling their partner than victims of KC. As Fissel [32,33] noted, the perceived severity of the cyberstalking behavior plays an important role in the decision to seek help. Therefore, victims’ perceived support is crucial in coping with the phenomenon. Regarding the presence of anxiety symptoms, 125 participants (58.1%) reported a state anxiety score that ranged from mild to high. State anxiety was defined as unpleasant emotional arousal in the face of threatening demands or dangers. A cognitive appraisal of the threat is a prerequisite for experiencing this emotion [52]. Thus, the majority of the respondents experienced anxiety symptoms in response to the fear triggered by the cyberstalker. In addition, the results of this study showed that 140 participants (62%) exhibited symptoms of trait anxiety. Trait anxiety is part of personality and reflects the existence of stable individual differences in the tendency to respond to threatening situations. The majority of respondents were activated even when there was no threat. They experienced the behavior of cyberstalkers in a continuous state of threat, in a seamless situation.

In addition, the results of this research show that there is a difference in the experience of cyberstalking whether the misconduct was committed by a UC or a KC. KC’s victims reported more about the consequences, fatigue, sadness, lack of trust in others, and anxiety symptoms than UC’s victims. These findings partially supported the first hypothesis of this study: cyberstalking experiences exacerbate physical and emotional symptoms, especially in KC’s victims. The results on depressive symptoms and anxiety showed no statistically significant difference between victims of UC and KC. As described above, victims of both types of cyberstalkers experienced negative emotions, including depressive and anxiety symptoms. To help victims, the consideration of the Modena Group on Stalking [53] might be useful to provide insight to practitioners who intervene: The proposal focuses on the emotional processing of the misbehaviors. Having experienced a change in their basic beliefs about the adequacy and safety of the environment in which they live, victims feel an extreme sense of vulnerability and fear of being attacked at any moment. Cognitive therapy could help restructure the pathological beliefs that threaten the victim’s functioning and allow them to develop a more realistic and acceptable conception of their sense of safety. If the stalking is still ongoing, the victim’s fears have a real basis, so the cognitive tools still need to be provided with consideration of the real security problem. It may also be useful to incorporate behavioral therapy interventions, such as gradual exposure and desensitization exercises, which can help to gradually resume previously abandoned activities and overcome anxiety symptoms. Victims may also benefit from support groups, where feelings of isolation are reduced, and a sense of mutual understanding and validation prevails. It is also important to involve key family members, including the partner (if any). They can often be a source of additional information that will enable victims to develop better strategies for dealing with the problem. They can also support the victim’s own need for safety [54].

The importance of social support for victims was evident from the coping strategies used. Victims of UC were more inclined than victims of KC to talk to their partners. This could mean that it is difficult for victims of KC to talk about their experiences for fear of shame or criticism [55]. In addition, as expected, victims of KC tended to use a great variety of strategies more than victims of UC. Specifically, victims of KC used alcohol and drugs more, consumed psychological substances more, purchased a weapon, and attempted to reason with the cyberstalker. Victims of UC were more inclined than victims of KC to block online contact and ask a social network administrator to intervene. Thus, the second hypothesis was only partially supported: Victims of unknown cyberstalkers were more inclined to tell someone about their experience; however, victims of KC used more diverse coping strategies than victims of UC. Using Podanà and Imriskova’s [37] classification of coping strategies, this research found that victims of KC tended to use more proactive and passive behaviors than victims of UC, while victims of UC tended to use more avoidance tactics than victims of KC. As suggested by Sgarbi [56], the decision to take control of the situation must be made by the victims. Intervention could be multifaceted, focusing on their personal safety and the safety of others (parents, friends, etc.). Therefore, in order to manage the emotional frame and/or resolve the situation, victims may engage in proactive behaviors as well as avoid or ignore the persecutor and seek external help from friends, family, police, etc. The goal could be to define a safety plan that adapts to the change in the misbehavior and to the effectiveness of the strategy. In fact, satisfaction or frustration with the strategies chosen could increase or decrease perceived fear.

### Strengths and Limitations

As far as we know, this is the first time that the experience of cyberstalking, its consequences, and coping strategies have been studied from the perspective of victims of UC and KC. Some suggestions emerged from this research that might help victims who have experienced cyberstalking by known and unknown perpetrators. Because the misconduct causes fear, it might be useful to have a safety plan, develop a variety of coping strategies, and tell someone about the experience of victimization. Social support could increase the chance of breaking through the isolation that characterizes the experience of victimization [57]. The ability to offer support in the form of friends, partners, relatives, etc., could be increased through educational campaigns on the topic: This could promote acceptance that being a victim is not a social stigma [58].

This study had some limitations. First, it was a cross-sectional study, and the sample was not randomly selected. Therefore, the results should be taken with caution and should not be generalized. In addition, because the sample was too small, we could not disaggregate the data by sex of the victim and the perpetrator. For future studies, it would be useful to consider the role of gender of the two actors involved in the phenomenon and analyze how it relates to the relationship between victims and perpetrators. In addition, there might be a bias related to socially desirable responding, i.e., the tendency to answer a questionnaire while presenting a positive image of oneself. We did not use a social desirability scale [59] in conjunction with the other instruments used in this study. To improve the validity of questionnaire-based research, it could be included in future studies. In addition, it is difficult to describe oneself as a victim, the misconduct, the consequences, and how the victims tried to deal with it [60]. One of the reasons for this difficulty is the fear that they will not be believed or that they will be thought to be persecutory or unable to defend themselves. In addition, victims may deny or minimize the episodes [61]. Denial and minimization are coping strategies that allow one to ward off unpleasant feelings and rationalize the other person’s (the cyberstalker) behavior. These strategies can work and help the victim ignore the situation and the potential dangers and continue to go about his or her activities and usual routine. Sharing the experience with another person can help them think more objectively about the situation and find a solution.

## 5. Conclusions

From this research came the need to intervene immediately and offer help to victims to deal with cyberstalking behavior. This help can be informal, e.g., through a partner, relatives, friends, and/or formal, e.g., through a counseling center and/or the police. The ability to seek help from formal services provides victims with an opportunity to protect themselves from the perceived social stigma associated with victimization. Because the behavior occurs in the online world, advertisements could be made on social media about how to deal with the misconduct, such as phone numbers and email addresses to contact the support service. This could allow victims to deal with the phenomenon and interrupt the cyberstalker and the negative feelings associated with their behavior.

## Figures and Tables

**Table 1:** Distribution of the cyberstalking behavior.

	Online Contact (*n* = 117)	Online Harassment (*n* = 73)	Online Unwanted Sexual Advances (*n* = 102)	Online Threats of Violence (*n* = 43)	Online Identity Fraud (*n* = 41)
Victim’s Gender					
Male	28%	25%	16%	41%	32%
Female	72%	75%	84%	59%	68%
Perpetrator’s gender					
Male	78%	84%	91%	64%	62%
Female	11%	2%	4%	17%	19%
Unknown	11%	14%	5%	19%	19%
Relationship with the perpetrator					
Strangers	35%	47%	55%	24%	56%
Friend/Acquaintance	40%	46%	39%	62%	37%
Partner/Ex-Partner	25%	7%	6%	14%	7%

**Table 2:** Physical and emotional symptoms: comparison between victims of UC and KC (percentage in brackets).

	UC (*n* = 115)	KC (*n* = 127)	*Χ* ^2^	*p*	*ϕ*
Physical Symptoms					
Weight change	9 (7.8%)	16 (12.6%)	1.19	n.s.	0.075
Loss/increase of appetite	26 (22.6%)	27 (21.3%)	1.75	n.s.	0.091
Sleep disorders	31 (27%)	39 (30.7%)	0.15	n.s.	0.027
Headache	27 (23.5%)	25 (19.7%)	0.93	n.s.	−0.067
Tiredness	23 (20%)	41 (32.3%)	3.88	0.049	0.137
Nausea	11 (9.6%)	15 (11.8%)	0.17	n.s.	0.029
Weakness	20 (17.4%)	2 (1.6%)	0.02	n.s.	0.009
Self-inflicted injuries	1 (0.9%)	4 (3.1%)	1.41	n.s.	0.082
Use of laxatives	-	2 (1.6%)	1.71	n.s.	0.091
Forced vomiting	2 (1.7%)	4 (3.1%)	0.36	n.s.	0.042
Injuries (caused by the stalker)	-	3 (2.4%)	2.53	n.s.	0.111
Panic attacks	17 (14.8%)	20 (15.7%)	0.015	n.s.	0.009
Emotional symptoms					
Suicidal thoughts	5 (4.4%)	9 (7.1%)	0.69	n.s.	0.057
Suicide attempt	3 (2.6%)	1 (0.8%)	1.34	n.s.	−0.080
Sadness	40 (34.8%)	64 (50.4%)	5.26	0.022	0.159
Anger	60 (52.2%)	77 (60.6%)	1.30	n.s.	0.079
Confusion	41 (35.6%)	56 (44.1%)	1.39	n.s.	0.082
Fear	39 (33.9%)	53 (41.7%)	0.94	n.s.	0.067
Lack of confidence in others	21 (18.3%)	50 (39.4%)	12.25	0.001	0.242
Aggressiveness	15 (13%)	29 (22.8%)	3.40	n.s.	0.128
Paranoia	34 (29.6%)	46 (36.2%)	0.718	n.s.	0.058
Irritation	53 (46.1%)	71 (55.9%)	1.06	n.s.	0.071
Agoraphobia	5 (4.4%)	7 (5.5%)	0.103	n.s.	0.022

Note. UC = unknown cyberstalker; KC = known cyberstalker. The total percentage can be over 100 because the participant could choose multiple consequences related to the cyberstalking experience.

**Table 3:** Depressive and anxiety symptoms: comparison between victims of unknown and known perpetrators (one-way ANOVA).

	UC (*n* = 115) *M* (*SD*)	KC (*n* = 127) *M* (*SD*)	*F*	*p*	*η* ^2^
Depressive symptoms (Range: 0–39)	4.25 (4.88)	5.34 (5.38)	2.534	n.s.	0,011
Anxiety symptoms: state inventory (Range: 20–80)	41.27 (11.48)	43.18 (11.70)	1.451	n.s.	0.007
Anxiety symptoms: trait inventory (Range: 20–80)	42.20 (10.32)	45.18 (11.04)	4.629	0.032	0.020

Note. UC = unknown cyberstalker; KC = known cyberstalker.

**Table 4:** Telling someone about the cyberstalking experience: comparison between victims of UC and KC (percentage in brackets).

	UC (*n* = 115)	KC (*n* = 127)	*Χ* ^2^	*p*	*ϕ*
Police	4 (3.5%)	4 (3.1%)	0.09	n.s.	−0.02
Parents/family	33 (28.7%)	35 (27.5%)	0.67	n.s.	−0.06
Friends	70 (60.9%)	83 (65.3%)	0.54	n.s.	−0.06
Partner	36 (31.3%)	25 (19.7%)	7.98	0.005	−0.22
Physician	1 (0.9%)	1 (0.79%)	0.03	n.s.	−0.01
Psychotherapist	7 (6.1%)	9 (7.8%)	0.01	n.s.	0.01

Note. UC = unknown cyberstalker; KC = known cyberstalker. The total percentage can be over 100 because the participant could choose multiple consequences related to the cyberstalking experience.

**Table 5:** Coping strategies: comparison between victims of UC and KC (percentage in brackets).

	UC (*n* = 115)	KC (*n* = 127)	*Χ* ^2^	*p*	*ϕ*
Collect evidence	40 (34.8%)	45 (35.4%)	0.05	n.s.	−0.015
Decrease use of Internet	17 (14.8%)	27 (21.2%)	1.31	n.s.	0.078
Have a safety plan	-	2 (1.6%)	1.75	n.s.	0.090
Increase social contact	35 (30.4%)	32 (25.2%)	1.32	n.s.	−0.079
Increase misuse of alcohol	3 (2.6%)	14 (11%)	5.90	0.015	0.165
Increase use of drugs	-	5 (3.9%)	4.37	0.037	0.143
Decrease social contact	1 (0.9%)	10 (7.9%)	6.303	0.012	0.171
Increase use of psychotropic substances	1 (0.9%)	5 (3.9%)	2.17	n.s.	0.101
Buy a weapon	-	5 (3.9%)	4.41	0.036	0.144
Try to reason with cyberstalker	15 (13%)	34 (26.8%)	6.08	0.014	0.168
Block the online contact	82 (71.3%)	76 (59.8%)	8.54	0.003	−0.200
Ask for an intervention by a social network administrator	39 (33.9%)	13 (10.2%)	22.05	0.001	−0.321
Ask for an intervention by a phone administrator	4 (3.5%)	4 (3.1%)	0.04	n.s.	−0.014
Change identity online	5 (4.4%)	5 (3.9%)	0.07	n.s.	−0.018
Contact the postal police	5 (4.4%)	4 (3.1%)	0.31	n.s.	−0.038

Note. UC = unknown cyberstalker; KC = known cyberstalker. The total percentage can be over 100 because the participant could choose multiple consequences related to the cyberstalking experience.

## Data Availability

Not applicable.
